# An adaptive geometric search algorithm for macromolecular scaffold selection

**DOI:** 10.1093/protein/gzy028

**Published:** 2018-11-08

**Authors:** Tian Jiang, P Douglas Renfrew, Kevin Drew, Noah Youngs, Glenn L Butterfoss, Richard Bonneau, Den Nis Shasha

**Affiliations:** 1 Computer ScienceDepartment, Courant Institute of Mathematical Sciences, New York University, New York, NY, USA; 2 Center for Genomics and Systems Biology, New York University Abu Dhabi, Abu Dhabi, UAE; 3 Center for Computational Biology, Flatiron Institute, Simons Foundation, New York, NY, USA; 4 Center for Systems and Synthetic Biology, Institute for Cellular and Molecular Biology, University of Texas at Austin, Austin, TX, USA

**Keywords:** enzyme design, foldamer, loop modeling, metal binding, octree

## Abstract

A wide variety of protein and peptidomimetic design tasks require matching functional 3D motifs to potential oligomeric scaffolds. For example, during enzyme design, one aims to graft active-site patterns—typically consisting of 3–15 residues—onto new protein surfaces. Identifying protein scaffolds suitable for such active-site engraftment requires costly searches for protein folds that provide the correct side chain positioning to host the desired active site. Other examples of biodesign tasks that require similar fast exact geometric searches of potential side chain positioning include mimicking binding hotspots, design of metal binding clusters and the design of modular hydrogen binding networks for specificity. In these applications, the speed and scaling of geometric searches limits the scope of downstream design to small patterns. Here, we present an adaptive algorithm capable of searching for side chain take-off angles, which is compatible with an arbitrarily specified functional pattern and which enjoys substantive performance improvements over previous methods. We demonstrate this method in both genetically encoded (protein) and synthetic (peptidomimetic) design scenarios. Examples of using this method with the Rosetta framework for protein design are provided. Our implementation is compatible with multiple protein design frameworks and is freely available as a set of python scripts (https://github.com/JiangTian/adaptive-geometric-search-for-protein-design).

## Introduction

In the past 15 years, protein design has advanced considerably in scale, accuracy and the variety of design tasks carried out by practitioners. Early successes in protein design focused on protein fold design (including novel folds) (Kuhlman *et al.*, 2003) and hyperstabilization of proteins (Dantas *et al.*, 2003). The redesign of protein–protein (Boyken *et al.*, 2016) and protein–DNA (Ashworth *et al.*, 2006) interfaces is a step towards functional rewiring of biological networks. More recently, protein engineers have turned toward the redesign of protein active sites and smaller functional patterns that demand sub-angstrom accuracy in the positioning of key side chains. Such works include both the engraftment of known active sites onto new scaffolds (Jiang *et al.*, 2008) as well as the engraftment of novel active sites (derived from quantum mechanical modeling of desired reactions) (Röthlisberger *et al.*, 2008) onto new scaffold proteins. In these enzyme design applications, active site patterns can become quite large—as residues involved in substrate binding, reaction mechanism and the surrounding environment may be considered. Enzyme design and related design tasks involving functional site or hotspot transplantation depend, in part, upon methods for matching a spatial pattern of chemical functional groups onto large libraries of potential scaffolds (proteins, nucleic acids or synthetic peptidomimetics, for example).

The earliest geometric matching applications in bioinformatics were aimed at matching whole substructures that indicated a likelihood of shared protein function or distant homology (Holm and Laakso, 2016). In many cases, these algorithms searched for contiguous regions and essentially functioned as the structural analog of sequence alignment algorithms (both gapped and ungapped). Applications included protein function prediction, analysis of protein structure prediction and evaluation of new algorithms (Nussinov and Wolfson, 1991; Fischer *et al.*, 1992; Bonneau *et al.*, 2002). Related work included innovative geometric hashing to extract 3D functional motifs from protein structures (Wallace *et al.*, 2008). In this work, we focus on geometric searches for biodesigns rather than prospecting or annotation.

Geometric searches developed for similar design tasks have used combinations of geometric hashing, side chain conformation libraries and other heuristics that have typically limited the number of functional elements in any given search pattern. Fleishman *et al.* computationally designed a protein to bind hemaglutinin (HA), targeting a conserved region on the stem (Fleishman *et al.*, 2011). They first identified the spatial positions of possible high-affinity (hotspot) residues by docking single amino acids onto the HA stem region and calculating a binding energy. Next, for residues predicted to have sufficient binding energies to HA, they built inverse rotamer libraries (i.e. rotamer distributions rooted at the side chain functional group rather than the backbone)—which served as anchor sites on which to dock protein scaffolds. The protein scaffolds themselves were selected from proteins not known to bind HA and were filtered for high shape complementarity with the HA target region. A low-resolution docking procedure was used to simultaneously optimize the HA scaffold binding energy as well as the scaffold’s ability to accommodate anchor residues. Scaffolds that showed geometric complementarity with the satisfied hotspot residues were used as the starting point for a second round of docking and design to optimize scaffold side chain positions surrounding the hotspot residues.

There are additional examples of geometric search-driven design on synthetic oligomeric foldamers and short peptidomimetic scaffolds. The objectives of the peptidomimetic design task may vary considerably: e.g. active-site mimicry, interface binding, metal binding or surface adhesion (Pacella *et al.*, 2013). The set of oligomeric scaffolds can provide protein-like side chain spatial armaments is quite diverse; examples include linear peptoids (Zuckermann *et al.*, 1992), oligooxopiperazines (OOPs) (Tošovská *et al.*, 2010), HBS helices (Chapman *et al.*, 2004), cyclic peptides (Bhardwaj *et al.*, 2016) and peptoids (Yoo *et al.*, 2010), β-peptides (Molski *et al.*, 2013) and hybrids thereof. A frequent aim is to mimic protein–protein interfacial hotspots in which a small number of side chains scaffolded by a single secondary structure element comprise a significant fraction of the binding energy (Watkins and Arora, 2015). In these cases, moving such side chain groups to a new, non-protein, scaffold with synthetically restricted backbone degrees of freedom and reduced atomic mass may be a viable route to inhibiting protein–protein interactions (PPIs). Lao et al. (2014) showed that by grafting four side chains from a restricted segment of sequence onto a four-subunit OOP scaffold creates low nanomolar inhibitors of two important PPIs (p53-MDM2 and p300-Hif1α). The first step in this work used a geometric search to dock the OOP scaffold into the binding site, such that side chain take-off angles were compatible with those the three hotspot residues (predicted to comprise the majority of the binding energy) in the experimental structure. After the geometric search instantiated a starting pose, the Rosetta design procedure (with modifications for both NCAA side chains and the OOP backbone) was used to optimize binding—resulting in low nanomolar inhibitors of both complexes. In both cases, the geometric match steps were based on expensive inverse rotamers searches, which limits the procedure to only small peptidomimetics.

Drew et al. (2013) previously demonstrated the incorporation of several non-peptidic backbone chemistries in the macromolecular modeling suite, Rosetta. There are many additional abiotic foldamer and peptidomimetic backbones (Guichard and Huc, 2011) that are amenable to such treatment. Determining the foldamer backbone (or hybrid chemistry) most compatible with a given interface is a potential bottleneck as the number of synthetically accessible scaffolds for biomimicry continues to increase.

Here, we describe a new method combining octrees (a data structure that maps regions of 3-dimensional space to nodes in a tree) and a novel adaptive search that grants a significant performance gain for the applications described above. Key innovations include the ability to weight interaction/pattern components by energy and the adaptive nature of the search, which both increases efficiency and allows for specification of error tolerance (per component of the template pattern) and number of mismatches. We pose the problem by describing a typical setup. We then describe our core algorithm. Finally, we describe applications to protein and peptidomimetic design tasks.

## Methods

### Problem setup

Given a library of molecular scaffolds, our method will find a suitable set of scaffolds to cause a set of target functional groups to be fixed in space relative to one another. We use the term *functional group* to indicate the terminal atoms of a side chain, i.e. those atoms whose position will remain fixed relative to one another during the rotation of the *χ* angles of the side chain. Examples would not only include the phenyl, imidozol and guanadinium groups of phenylalanine, histidine and arginine, respectively, but also the four terminal carbons of leucine (Cβ, Cγ, Cδ1, Cδ2) and the hydrogens that branch from them. A *molecular scaffold* is defined generally as any molecule from which designable side groups could branch.

A given scaffold will typically have varying degrees of freedom and these degrees of freedom will therefore define that scaffold’s ability to accommodate fixed functional groups. Practically, different scaffolds will have different degrees of flexibility at different positions and this will drive our definition of allowable error of matching. For a peptide, the predominant degrees of freedom are the *φ* and *ψ* angles of the backbone and *χ* angles in the side chains. Peptidomimetic scaffolds will have different degrees of freedom. For example, in peptoids we must also consider the *cis*/*trans* state of the preceding-*ω* angle, which potentially allows for greater diversity of side chain Cα–Cβ bond vectors for a given sequence. Alternatively, an OOP scaffold, which has cyclic constraints between neighboring residues, is theoretically much more restricted in its ability to accommodate fixed functional groups but also has a reduced entropic cost upon binding a target.

Our approach to interface design is a two-step process. In the first step, we consider the most influential energies and conduct an efficient geometric search to eliminate all the impossible designs. In a second step, designs that passed the quick initial screening are further refined using the Rosetta suite (Leaver-Fay *et al.*, 2011), potentially introducing additional mutations. This two-step process efficiently saves all the time that the majority impossible designs would take to be evaluated by Rosetta.

**Fig. 1:**
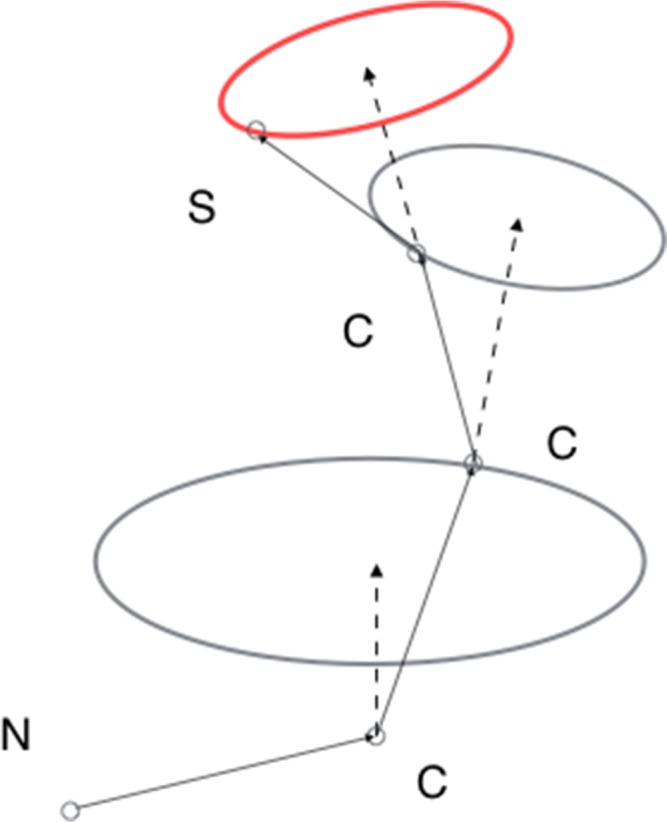
An example of the manifold generation of the side chain N-C-C-C-S. The manifold constitutes of all possible positions of S through rotations of each bond with fixed bond angles.

In the first step, since we consider only the most optimal bond angles, the problem is reduced to the following abstract math problem. We consider the binding interface configuration as a polygon whose vertices are the binding nodes. For each side chain, all the possible positions of one binding node (often the end node of the side chain) form a manifold in 3D obtained by fixing all the bond angles to the optimal ones and sample possible rotations of dihedral angles (Fig. 1). Thus, the task is to find (at least) one potential binding node from each manifold such that they form a ‘desirable configuration’ should such a ‘desirable configuration’ exist (and later possibly check that all dependent nodes do not physically collide with every other node).

Let $\langle P=P_{1},P_{2},\cdots ,P_{n}\rangle$ be the target polygon. In this paper, all polygons are denoted by putting angle brackets around their ordered vertices. We define the error ε of a configuration or a polygon $\langle S=S_{1},S_{2},\ldots ,\;S_{n}\rangle$ by its distance to the target configuration P defined as $\max_{1\leq i,j\leq k}\left|\;P_{i}\;P_{j}-S_{i}S_{j}\right|.$

Following the standard notation, we use capital letters to denote points in space and we write AB to denote the length of the line segment joining the points A and B. Let $\varepsilon_{T}$ be the maximum error we allow to account for the smaller energies we are ignoring and errors due to the discretization of the manifolds. If $\varepsilon <\varepsilon_{T}$, we call the configuration or polygon S a ‘desirable configuration’. Therefore, the problem of the peptoid design is to select the best side chain and backbone constitutions such that there exists a desirable binding configuration while maintaining a low-energy state.

### Adaptive geometric search algorithm

We employ octrees as the core data structure for our algorithm (Berg *et al.*, 2008). A cubic volume, with sides of length *l*, centered on a point *p*, can be subdivided into eight cubes with sides of length *l*/2, that share *p* as a vertex. Each of these eight cubes can be further subdivided into eight more cubes each with side of length *l*/4, and so on. This decomposition of 3D space lends itself to a tree-like representation called an octree. Thus, octrees are tree structures whose nodes correspond to 3D cubes embedded in a hierarchically subdivided overall 3D space and each deeper level of the tree describes a successively smaller volume of space. Each node has eight ‘children’ nodes obtained by subdividing each side of the cube by the middle in the *x*, *y* and *z* dimensions. All the 3D objects, in our case, points in 3D, are stored in the leaf nodes. Octrees have various stopping criteria to prevent the tree from splitting into forever smaller cubes, including thresholding based on the number of 3D objects in a node, i.e. the octree splits only if the nodes contain more than a certain number of 3D objects. For our problem, these 3D objects are simply points in the 3D space and the stopping criterion is the minimum cube length *l*_*s*_. That is, the octree splits a node only if its corresponding cube has sides of length at least 2*l*_*s*_. Moreover, all empty nodes, i.e. nodes whose corresponding cubes contain no points, are discarded.

To find desirable configurations, first we sample all possible positions of a functional group, or binding node by fixing all the bond angles to the optimal ones and sample possible rotations of dihedral angles (Fig. 1). This process forms a manifold for each functional group. Then the algorithm builds octrees using sampled points from each manifold and the tree stops branching at the leaf cubes of length at least *l*_*s*_. Next, the algorithm compares every two octrees at a time by testing the necessary and sufficient conditions on their corresponding nodes and searching adaptively only down the pairs of nodes that pass the necessary condition (see below). We call a pair of nodes (and the corresponding cubes) that pass the necessary condition a ‘possible pair’. The algorithm finds all the possible cube pairs at each tree level until it ends up with the set of all possible pairs of leaf cubes. Then it tests the sufficient condition on the possible pairs of leaf cubes to determine whether to accept or reject all the pairs of points inside them. At the end, all the pairwise desirable cubes are combined through a matrix product to identify desirable *n*-tuples or ‘desirable configurations’.

### Establishing necessary and sufficient conditions for matching

Our overall strategy is to enumerate all possible residue positions (when there is a choice on the particular scaffold) and amino acid assignments to these residues and then to use the adaptive geometric algorithm to determine whether the resulting functional groups, or binding nodes, at those positions have the proper geometry. Thus, the adaptive geometric algorithm is the ‘inner loop’ of the computation with the ‘outer loop’ being all possible residue positions and amino acid assignments. For this inner loop to be efficient, it must swiftly filter away impossible geometries (Theorem 1 below) and identify promising ones (Theorem 2 below).

Mathematically, the adaptive geometric algorithm efficiently searches for a certain *n*-polygon among *n* sets of points in 3D space given an error tolerance and an approximation margin. This general scheme is required for all the applications introduced above and evaluated in the Results section. Given a target polygon $P=\langle P_{1},\;P_{2},\ldots ,\;P_{n}\rangle ,$ a tolerance $\varepsilon_{T}\geq 0$ and one edge $(P_{i},\;P_{j}),$ let $C_{i},C_{j}$ be two non-empty cubes with size *l* and the distance between their centers *d*, where i,j∈{1,2,…,n},i≠j. Then we have the following theorems that help us determine which cubes could possibly contains pairs of points whose line segment matches that edge. That is, the theorems provide acceptance and rejection criteria for pairs of cubes from different trees (which correspond to different manifolds where each manifold corresponds to, for example, a take-off residue from a backbone). The first theorem provides a rejection criterion.

**Theorem 1**

*If*$d<P_{i}P_{j}-\varepsilon_{T}-\sqrt{3}l$*or*$d>P_{i}P_{j}+\varepsilon_{T}+\sqrt{3}l$, *then there are no pairs of points*$\left(G,\;H\right)\in C_{i}\times C_{j}$*such that*$\left|GH-P_{i}P_{j}\right|\leq \varepsilon_{T}$.

Theorem 1 suggests a ‘necessary condition’ for any two cubic regions on the same level of the trees to contain any desirable pairs of points (at distance $P_{i}P_{j}$). We are going to refer to the condition defined in Theorem 1 as ‘Necessary Condition 1’ in the sequel. If two cubes do not satisfy the conditions of this theorem, no pairs of points from them could possibly match the edge $\left(P_{i},\;P_{j}\right)$ and will be rejected. That is why we consider this to be a rejection condition for pairs of cubes. By contrast, we have the following ‘Sufficient Condition 2’ for all pairs of points from two leaf cubes to be desirable (an acceptance condition).

**Theorem 2**

*If*$P_{j}-\varepsilon_{T}-\sqrt{3}l\leq d<P_{i}P_{j}+\varepsilon_{T}-\sqrt{3}l$, *then all pairs of points*$\left(G,\;H\right)\in C_{i}\times C_{j}$*satisfy*$\left|GH-P_{i}P_{j}\right|\leq \varepsilon_{T}$.

Notice that the condition of Theorem 2 can hold only when $P_{i}P_{j}-\varepsilon_{T}+\sqrt{3}l\leq d<P_{i}P_{j}+\varepsilon_{T}-\sqrt{3}l$, or when $l\leq \varepsilon_{T}/\sqrt{3}$. Because the leaf cubes of the octrees must have length $l_{T}\leq 2l_{s}$, we require $l_{s}\leq \varepsilon_{T}/(2\sqrt{3})$.

**Fig. 2:**
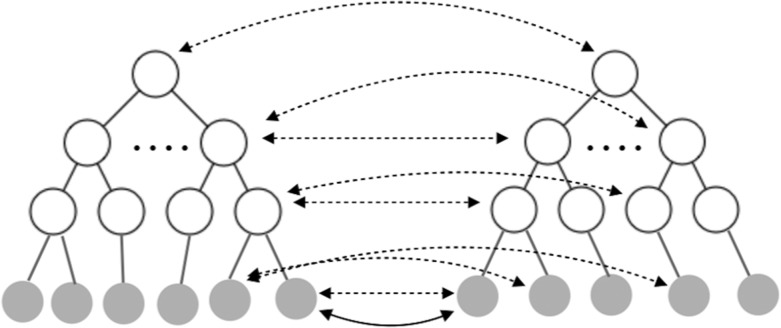
An illustration of the adaptive search between two octrees. Dotted lines point out the possible cube pairs on each level that pass the Necessary Condition 1. Solid lines link the desirable leaf cube (gray nodes) pairs that pass the Sufficient Condition 2.

Let *t*_*i*_ be the octree generated from manifold $A_{i}\;\mathrm{for}\;i=1,\;2,\;\ldots ,\;n$. Algorithm 1 gives the pseudocode of the adaptive geometric search algorithm. Figure 2 illustrates the algorithm graphically.

**Algorithm I.** 

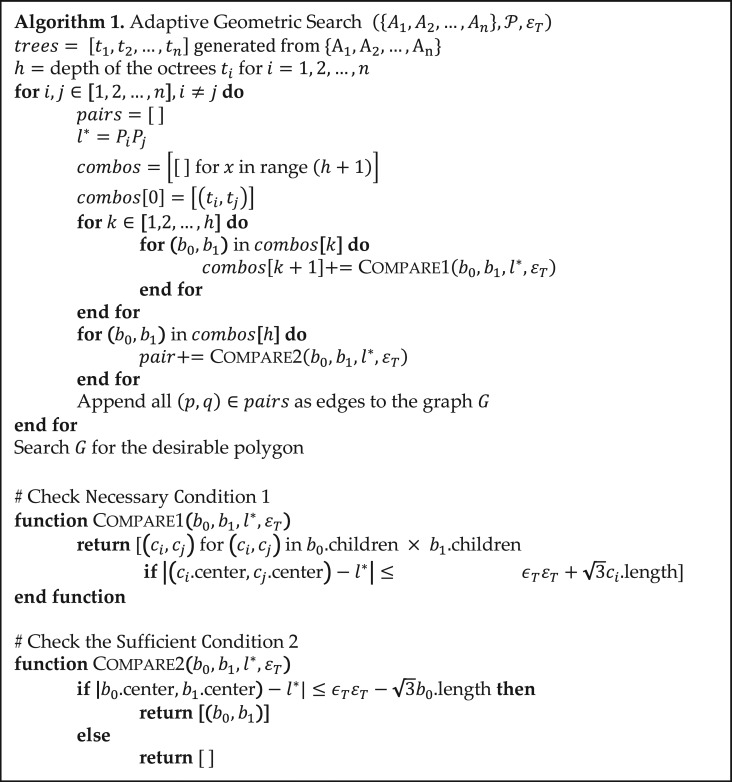

### Algorithmic complexity

The adaptive geometric search algorithm has three parts, building the octrees, adaptively searching every two octrees and the graph search. Let *N* be the number of sample points from each manifold. For convenience, we build all octrees with the same initial cube length *l*_*0*_. The time complexity of building an octree with initial cube length *l*_*0*_ and minimum cube length *l*_*s*_ is $O(\log_{2}\left(l_{0}/l_{s}\right)N)$.

Next, we compute the time complexity of the adaptive search between any two octrees (without loss of generality) called *t*_1_, *t*_2_. Let the corresponding polygon edge length be $l^{⁎}$.

**Theorem 3**

*If we set*$l_{s}=\eta \frac{\varepsilon_{T}}{4\sqrt{3}}$*for any*0<η<1, *then the adaptive geometric search (Algorithm 1) returns all the pairs of points whose distances are within the set*$\left[l-\left(1-\eta \right)\varepsilon_{T},\;l^{⁎}+\left(1-\eta \right)\varepsilon_{T}\right]$, *and some but possibly not all the pairs of points whose distances are within the set*$\left[l^{⁎}-\varepsilon_{T},\;l^{⁎}-\left(1-\eta \right)\varepsilon_{T})\cup (l^{⁎}+\left(1-\eta \right)\varepsilon_{T},\;l^{⁎}+\varepsilon_{T}\right]$.

**Proof**

See Appendix A.

**Lemma 4**

*Set*$l_{s}=\eta \frac{\varepsilon_{T}}{4\sqrt{3}}$*for some*0<η<1. *Then for any cube C*_1_*in an octree t*_1_, *there are at most*$\frac{4\pi }{3}\left(3\sqrt{3}+2\frac{4\sqrt{3}}{\eta }\right)(3{\left(\frac{l^{⁎}}{l_{s}}\right)}^{2}+{\left(\frac{3\sqrt{3}}{2}+\frac{4\sqrt{3}}{\eta }\right)}^{2})$*cubes*$C_{2}$*on the same level from another octree t*_2_*such that*$\left(C_{1},\;C_{2}\right)$*are possible pairs, that is, they satisfy the Necessary Condition 1.*

**Proof**

See Appendix A.

**Theorem 5**

*Recall that*$l_{0}$*denotes the initial cube length and the minimum cube length*$l_{s}=\eta \frac{\varepsilon_{T}}{4\sqrt{3}}$. *Le*t $n_{m}$*be defined as in Lemma 4. Then the time complexity of the adaptive search part of Algorithm 1 is*$O\left(\frac{1}{\eta^{6}\varepsilon_{T}^{5}}\right)$.

**Proof**

See Appendix A.

The last part of the algorithm is a graph search. Let $s_{ij}$ be the number of possible leaf cube pairs that also passed Sufficient Condition 2 between octrees $t_{i},\;t_{j}$ for i,j∈[1,2,…,n],i<j. We view the leaf cubes as vertices and possible pairs of them as undirected edges in the graph. If we want to produce all the desirable *n*-tuple cubes, then by induction it is easy to see that the upper bound on the time complexity is $C\left(S\right)=O\left(\prod_{1\leq i<j\leq n}s_{ij}\right)$.

In practice, we can probably do much better. Consider building a directed graph by giving directions to the edges to form an *n*-cycle of groups of cubes from $t_{1},\;t_{2},\ldots ,\;t_{n}$. Finding strongly connected components in this directed graph first would in most cases greatly reduce the search space at only a linear cost $O\left(\sum_{1\leq i<j\leq n}s_{ij}\right)$. For space reasons, we skip the algorithm details here.

In summary, we state the total time complexity of the algorithm as follows.

$$\begin{array}{c} \;O\left(n\;\log_{2}\left(\frac{l_{0}}{l_{s}}\right)N+\frac{n^{2}}{\eta^{6}\varepsilon_{T}^{5}}+\prod_{1\leq i<j\leq n}s_{ij}\right)\\ =\;O\left(n\;\log_{2}\left(\frac{4\sqrt{3}l_{0}}{\eta \varepsilon_{T}}\right)N+\frac{n^{2}}{\eta^{6}\varepsilon_{T}^{5}}+\prod_{1\leq i<j\leq n}s_{ij}\right)\\ =\;O\left(n\;\log_{2}\left(\frac{l_{0}}{\eta \varepsilon_{T}}\right)N+\frac{n^{2}}{\eta^{6}\varepsilon_{T}^{5}}+\prod_{1\leq i<j\leq n}s_{ij}\right). \end{array}$$

In practice, we usually search for a triangle or a four-sided polygon as the target polygon, i.e. n=3 or 4. When n=3, depending on the parameters $\eta ,\;\varepsilon_{T}$ and *N* the computation time varies but all three terms in the complexity formula (above) are typically of the same order. When there are large numbers of possible pairs s_*i*_’s and/or n=4, the term C(S) in the last term of the complexity formula (4.1) becomes the dominating term. The number of results *s*_*ij*_’s can be further reduced when we take optimal dihedral angles instead of uniform sampling from [0,2π]. Full proofs of the above theroms and lemmas can be found in the supporting information.

## Results

Our algorithm can be applied to many different problems in macromolecular modeling and design. It efficiently solves the problem of searching for a certain *n*-polygon among *n* sets of points in 3D with error tolerance $\varepsilon_{T}$ and an approximation margin η. We present three use cases where our algorithm’s improved efficiency (run times that are in some cases many thousands of times faster than previous approaches) improves the scaling of the overall task, enabling the use of larger template/target structural patterns.

### Scaffold matching: designing OOPs to inhibit MDM2-p53 interface

**Fig. 3:**
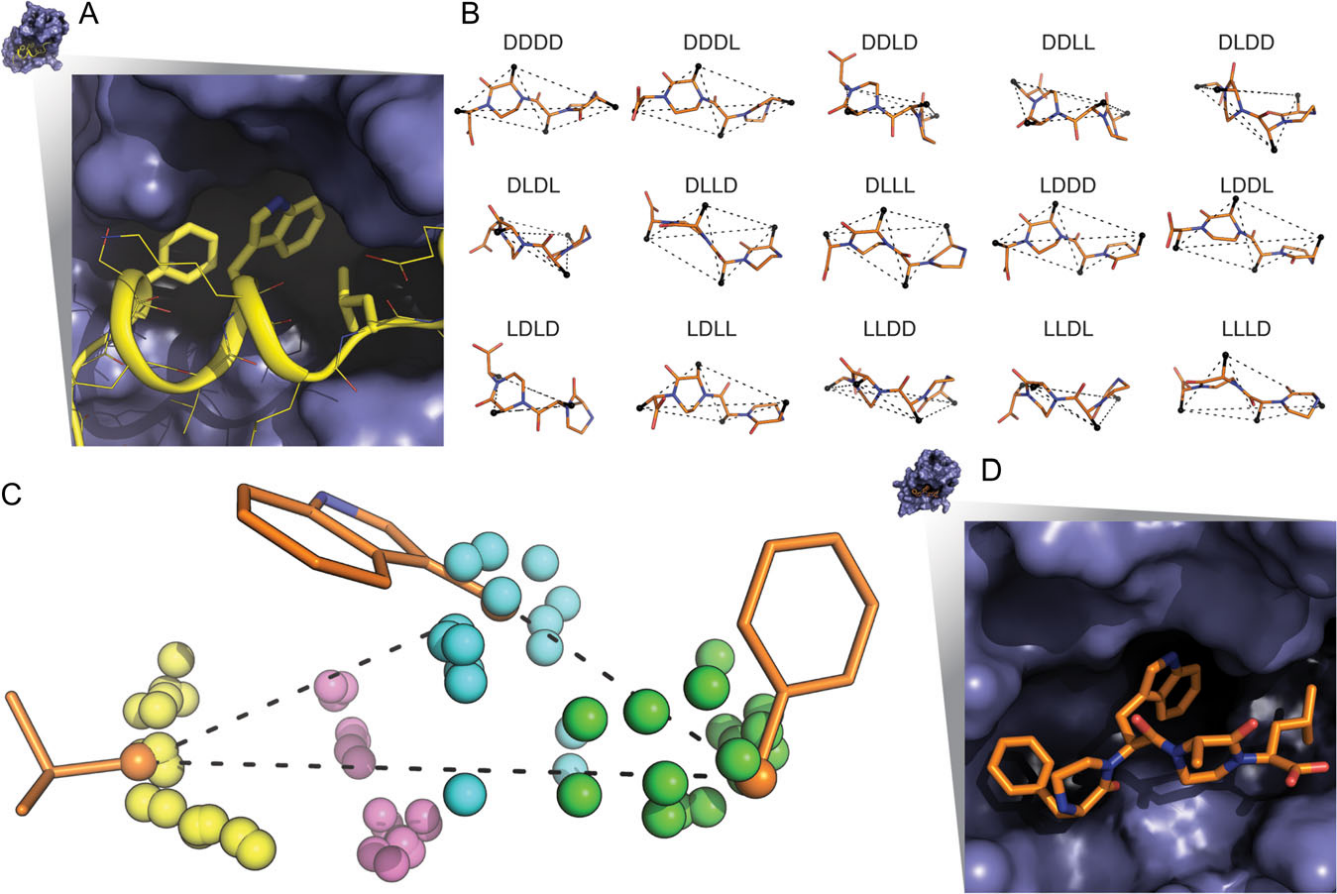
(A) The P53 (yellow) and MDM2 (blue) interface showing phenylalanine, tryptophan and leucine hotspot residues. (B) Fifteen of the 16 OOP backbone scaffolds fit to hotspot residue stubs. Scaffolds combinatorically sample the l or d enantiomers of the four residues that comprise the OOP scaffold. Each backbone has four Cβ atoms (black spheres) and thus four possibly matching triangles indicated by dashed lines. (C) The P53 hotspot residue stubs (orange). In this work, each hotspot residue has two *χ* dihedral angles resulting in a single fixed Cβ (orange spheres) triangle (dashed lines). Hotspot residues with additional *χ* angles would produce multiple triangles. Colored spheres show potential Cβ atoms from the OOP scaffolds for the first (green), second (cyan), third (magenta), fourth (yellow) residues in the scaffold. (D) The LLLL-OOP scaffold (orange) designed by Drew and coworkers and correctly identified by the algorithm bound to MDM2 (blue).

PPIs mediate many cellular functions and a small number of residues that make significant contributions to the binding affinity of the PPI (deemed ‘hotspot’ residues) in turn underlay these protein interfaces. Design tasks aimed at protein interfaces abound, as discussed above Fleishman *et al.* designed an influenza HA binder. Interest in using smaller, easy to synthesize, non-protolyzable macromolecules (called foldamers) as potential therapeutic candidates continues to rise as these systems become more synthetically (and computationally) accessible to a broader community. Foldamer backbone chemistries abound and finding a foldamer backbone type that is well matched to a particular set of interface hotspot residues interface will prove to be a future challenge. Here, we recapitulate an OOP foldamer designed by Drew and coworkers that mimics P53 and can disrupt the P53/MDM2 interaction (Fig. 3), which relies on three hotspot residues on P53 that constitute the majority of the binding affinity for MDM2 (Fig. 3A).

There are two parts of the algorithm. In Step 1, we search through all possible backbones for a matching triangle to the target triangle. In Step 2, for every match result from Step 1, the connecting atom’s bond angles are checked against the optimal bond angle. If a match passes Step 2, it is returned as a final result. Otherwise, we continue the iteration in Step 1.

The target triangle is made up of Cβ’s of the hotspot residues (Fig. 3C). The algorithm simply searches through the possible take-off position combinations, four triangles in this example (Fig. 3B), from every backbone for a match in shape within the error bound. Notice that in this case, all Cβ atoms are fixed due to the short lengths of hotspot residues. With longer hotspot residues, there will be a manifold of all the possible Cβ atoms for each hotspot residue. For every possible triplet of take-off positions, there are eight possible d and l-enantiomers. So, for each of these 32 possibilities, we apply adaptive geometric search to find all matches.

Once we have the matching shapes, we calculate the corresponding matrices *R*’s of rotation and translation such that after applying these transformations *R*’s backbones are connected onto the hotspot residues at atoms Cβ’s. Finally, we check if the bond angles at the connecting atoms (e.g. N, Cα and Cβ for leucine) are within some error bound to the optimal bond angles (indicated by the CheckAngle function in Algorithm 2).

**Algorithm 2.** 

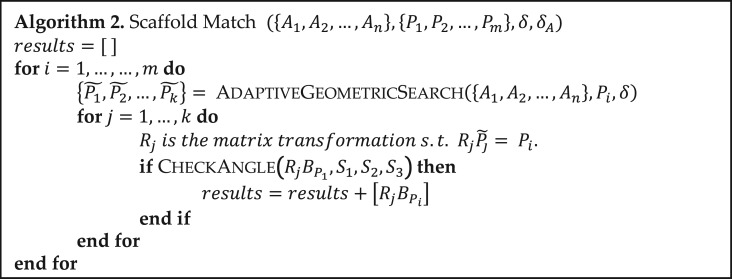

**Fig. 4:**
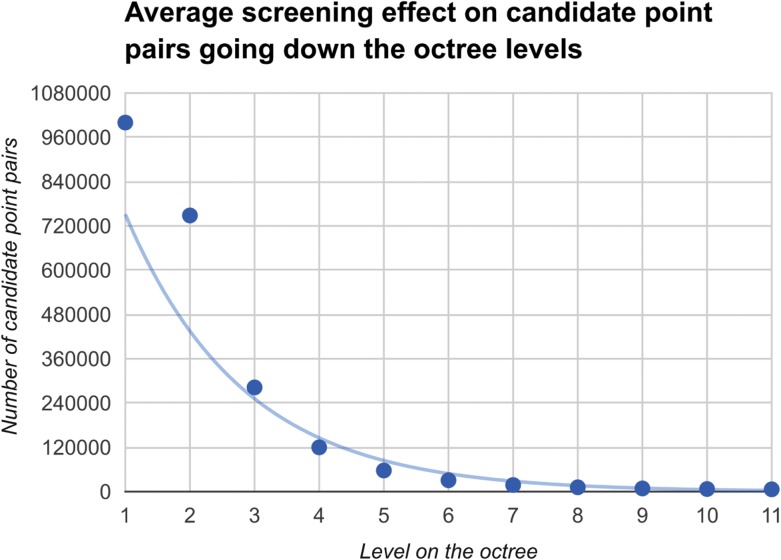
Averaging over 60 runs on different octree pairs, we show the number of candidate point pairs goes down at least exponentially as we select only those pairs of cubes that pass the Necessary Condition 1. The best exponential fit to the data is shown.

Let $A_{i}$ be the manifold of possible positions of the connecting atom on the *i*th hotspot residue. For example, in Fig. 3C points in colors are sampled from manifolds $A_{1},\;A_{2}$ and $A_{3}$ respectively. Let $P_{j}$ be the *j*th polygon of the backbone take-off position combination and for example, there are 4 × 17 of them in Fig. 3B. Let $B_{P}$ denotes the atoms’ position matrix corresponding to the backbone where the target polygon P comes from. Let $S_{i}$ denotes the atoms’ position matrix for the *i*th residue. Let δ be the distance error bound and $\delta_{A}$ be the angle error bound. Then we describe in pseudocode Algorithm 2. In the adaptive geometric search part of Algorithm 2, the possible candidate pairs are screened out at least exponentially fast as we search down the octrees (Fig. 4). Let C denotes the time complexity for adaptive geometric search. Recall that m is the number of target polygons from backbone take-off site combinations. Then the time complexity of the scaffold matching algorithm is O(Cm).

## Algorithms

**Fig. 5:**
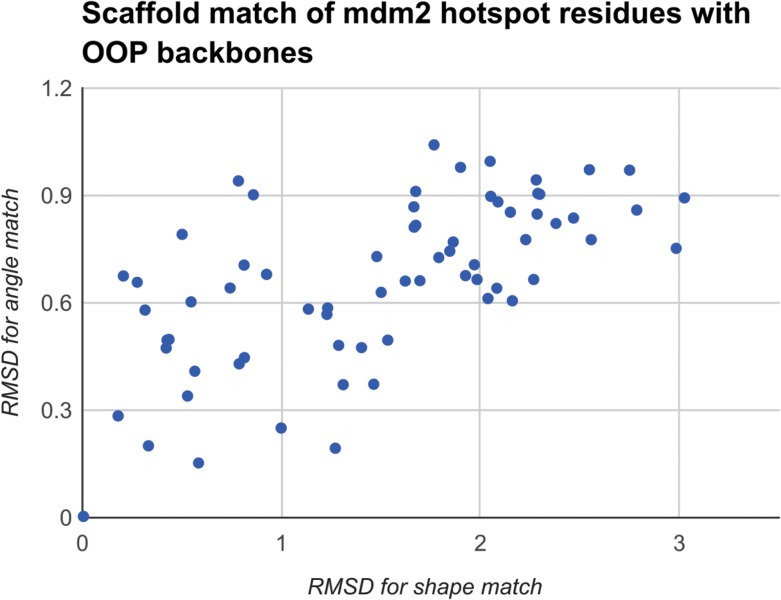
RMSD of all possible OOP backbones matches with the hotspot residues’ side chain positions. The candidate at the origin is a perfect match for both (shape and angle) to the hotspot residues we aim to minimize (use as a template for design) and is analogous to a template used in previously reported successful experimental designs.

In the search process, we scored all the possible matches by the root-mean-square deviation (RMSD) values for both shape match and angle match in Fig. 5. Our algorithm picked the candidate at the origin (this being identical to the correct conformation that led Lao *et al.* to low nanomolar inhibitors of this interface). In Fig. 3D, we show this best design for the OOP backbone of the hotspot residues. The run time for the initial geometric search (step on in this design protocol) is 0.02 to 0.12 s, whereas running the same design and producing the same results using the previously described Rosetta codes (the scripts from Lao *et al.*) takes ~18 min (a speedup of >9 000-fold).

### Peptoid design: design of new metal binding sites

Proteins and other macromolecules often coordinate metal ions to aid conformational stability or carry out chemical reactions. Proteins that bind Zn^2+^ ions often use four residues (most often histidine, cysteine or aspartic acid) to coordinate the zinc ion in a tetrahedral arrangement (Hsin *et al.*, 2008). We next tested our algorithm by designing a peptoid for capturing zinc ions. The binding sites we target in this example are three sulfur atoms lying on the vertices of an equilateral triangle.

**Fig. 6:**
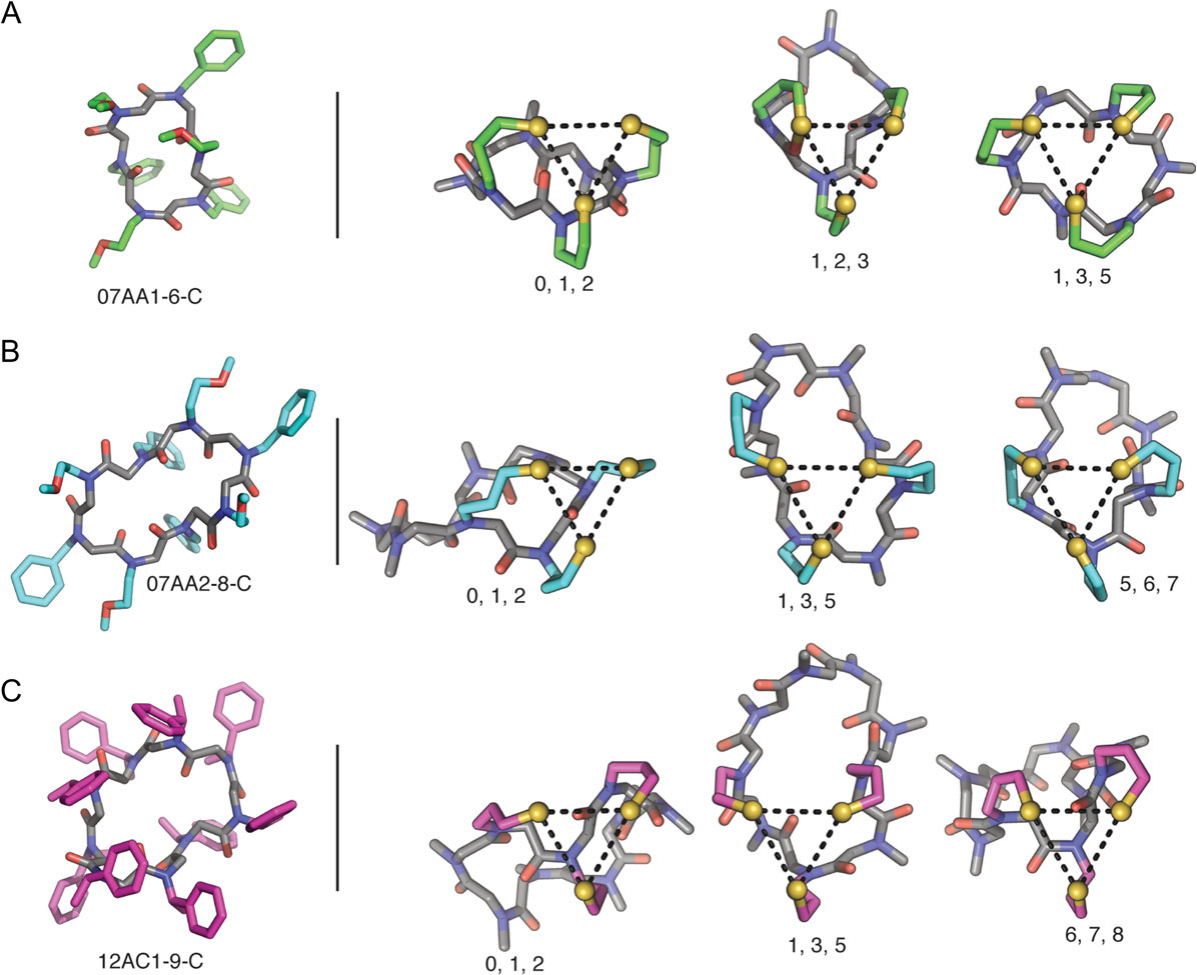
Experimentally determined peptoid macrocycle structures and representative examples of low-energy matches for the (A) 07AA1-6-C (B) 07AA2-8-C and (C) 12AC1-9-C peptoid macrocycle backbone scaffolds. Numbers under representative examples indicate residue position of 3-aminopropyl-1-thiol side chain.

The search space includes 6-mer, 8-mer and 9-mer scaffolds (peptoid data bank codes 07AA1-6-C, 07AA2-8-C (Shin *et al.*, 2007) and 12AC2-9-C (Butterfoss *et al.*, 2012), respectively) as the backbone and 3-aminopropyl-1-thiol groups as side chains of residues (Fig. 6). Low-energy matches were identified for each scaffold and commonly found to be comprised of alternating residue positions or sequential positions on the narrow end of the macrocycle.

We sampled eight dihedral angles per residue with different lengths of side chains (*n* = number of carbon atoms), different error values. We recorded the run time to find the first valid target polygon on Intel Core 3.5 GHz (Table I).

**Table I.** Run times for matching different geometric representations of metal-binding sites to a library of peptoid (peptidomimetic) scaffolds. Run times are shown in seconds for runs computed on an Intel Core 5 3.5-GHz processor. Run times are shown for three classes of binding site pattern and for various user-defined settings (corresponding to different allowable error and approximation ranges in atomic units)

err	0.05			0.1			0.5		
*η*	0.1	0.2	0.3	0.1	0.2	0.3	0.1	0.2	0.3
General triangle:									
*n = 2*	2.69	2.42	2.49	2.92	2.40	2.50	7.81	6.93	6.97
*n = 3*	76.64	73.56	67.87	106.96	101.12	91.04	1518.47	1357.48	1178.47
Equilateral triangle:									
*n = 2*	5.43	5.12	4.78	5.41	4.47	4.37	5.29	4.10	3.93
*n = 3*	181.04	161.34	139.75	175.37	151.46	124.03	272.62	223.75	176.27
General 4-gon:									
*n = 2*	7.63	7.06	7.15	7.75	7.33	7.47	9.67	8.67	8.82
*n = 3*	224.16	218.69	209.19	271.98	254.81	235.12	3262.67	2780.21	2079.59

### Loop modeling

**Fig. 7:**
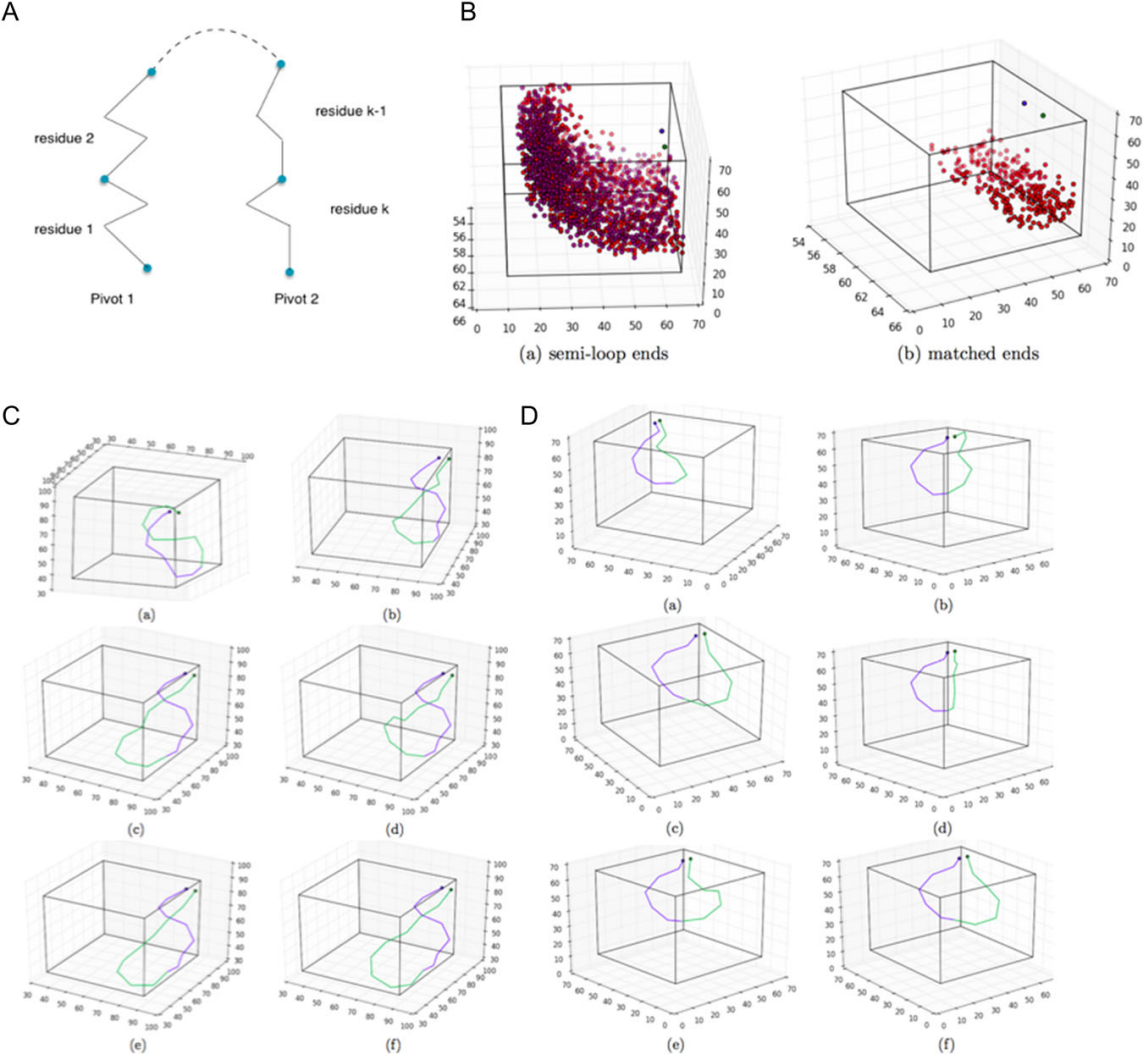
(A) Illustration of the loop closure algorithm set up. (B) (a) The blue and green points are pivot 1 and pivot 2. Red points are ends of the semi-loop growing from pivot 1 and purple points those from pivot 2. (B) (b) Matched ends in position and bond angle. For clarity only every 100th points are depicted in both figures. (C) Blue and green colors represent two semi-loops of six residues. Each line segment corresponds to one residue, connecting the first and last atoms of the residue. (D) Blue and green colors represent two semi-loops of eight residues and nine residues, respectively. Each line segment corresponds to one residue, connecting the first and last atoms of the residue.

Accurately modeling protein loops is a difficult problem due to their flexibility due to their and lack of regular structure (Mandell *et al.*, 2009). Computational modeling of loops generally involves defining the loop region (the residues that are flexible), anchor or pivot positons residues beyond which the protein structure remains fixed, and a cut point position at one of the residues in the loop region that splits the loop into two parts. A structural perturbation is made to one side of the loop—resulting in a break in the loop at the cut point—and the loop modeling protocol modifies the conformation of the other side of the loop in an attempt to close the break (Stein and Kortemme, 2013). The abstraction of the problem can be described as follows. Given two fixed points in 3D called pivots and two vectors (the take-off vectors), construct the loop from pivot 1 to pivot 2 with *k* residues with the type N-Cα-C such that the loop has a low energy and it fits in the designated space (Fig. 7A). Biologically relevant loops can vary greatly in length; the H3 CDR loop in human antibodies, for example, can vary from 5 to 26 residues (North *et al.*, 2011). The difficulties of the problem using a direct computation stem from exponential growth in the number of possible loop conformations as a function of loop length, *k*. We divide the loop into two semiloops by the midpoint or the closest point to the midpoint between two residues. The designated space where the loop resides within can be discretized into cubes of a certain size. We precompute all conformations of a single residue and store the resulting angles and x,y,z coordinates after discretization and encoded as a unique integer. Then we compute and store the table where two residue conformations can connect appropriately, that is, the end atom of one residue and the beginning atom of the other residue lie in the same cube and the two bonds form an angle within the error bound from the optimal bond angle. Now using the precomputed residue conformations and matching table, we develop the two semi-loops. Let the number of residue conformations be $M_{r}$ and the number of cubes in the lattice space $M_{c}$. After developing each residue, we collapse the end positions that fall into the same cube and sharing the same last bond angle and store all intermediate results for the purpose of producing final results in backtracking. After the two semi-loops are developed, we have the end atoms of both sides and their spatial intersections. The angles are checked to eliminate from the intersection cubes those that deviate outside the error bound from the optimal bond angle there. Starting from the matched cubes in the middle of the loop, now we backtrack in both sides to the pivots and produce as many results as desired (effectively allowing for efficient sampling of a large number of constraint-compliant loop designs). In the first experiment, we computed a 12-residue loop, developing 1000 conformations for each residue and 121 by 121 by 121 cubes in the designated space, setting cube length to 0.1 and maximum bond angle errors to within 0.2 rad. On a 1.3-GHz Intel Core M with 8 GB memory, our algorithm ran a total of 3.6 min to produce the first result (Fig. 7C). The development of each semi-loop took 82 s and the matching in the middle took 20 s. Keeping the number of conformations per residue, error bounds and the cube size, we enlarge the number of cubes to 171 by 171 by 171 to compute for 17-residue loops. On a 2.0-GHz Intel Xeon E5-2620 CPU with 128 GB memory, our algorithm ran a total of 35.5 min to produce the first result (Fig. 7D). The development of each semi-loop took 11 min and the matching in the middle took 28 s.

## Discussion

We have presented an adaptive method for finding matches between target geometric patterns (that represent protein and peptidomimetic design goals) and scaffolds (which can serve as the biosynthetic or organic synthesis method for positioning side chains in the desired/target geometry). In the protein, enzyme, and peptidomimetic design communities, such geometric search problems are increasingly becoming limiting steps in design processes. This trend will increase as we scale to larger target patterns and as we compare to growing databases of proteins, peptidomimetic structures and other scaffolds. Thus, improving the speed of geometric function scaffold search algorithms makes a substantive contribution to biomimetic design. We have tested our adaptive octree method in two realistic design settings (each one adapted from a recent publication using geometric target-scaffold or geometric matching) and, in each case, we were able to speed up the required calculation by 100 to 10 000-fold over the best previous methods. These speedups allow us to guarantee scaling and run times in a wide variety of design tasks. In addition, our algorithm allows for an explicit specification of allowable error rates and mismatches (built into both the search and the initial construction of the core octree data structure). Future work could include providing a better interface to the specification of error and allowable mismatches, resulting in a mismatch tolerant geometric search (akin to gaps in sequence alignments). Another area for future work would be to adapt our geometric search to a multiple-alignment setting, allowing us, for example, to seed a search and subsequently update the parameters of the search to reflect families of discovered sites on proteins. This would provide an algorithmic framework for iterative construction of functional sites on proteins that would be informed (in a data-driven manner) by geometric variation across discovered functional sites.

An immediate advantage of our improvement in computational efficiency is that it expands, by improving scaling, the range and types of peptidomimetic and protein scaffolds that can be explored. For example, our method dramatically increases the maximum pattern (active site to match to potential scaffolds) that can be engrafted via matching. This is important for enzyme design and catalysis design, as full sites that include substrate binding and catalytic sites can include large numbers of side chains (large numbers of component vectors in the template/starting geometric pattern to be matched/searched) (Jiang *et al.*, 2008; Röthlisberger *et al.*, 2008). The design of protein-binding sites can also involve large target patterns that challenge previous methods. Our work here opens the door to a more efficient approach to selecting scaffolds for designing these larger surface patterns. Our examples here show (presented above and as supplemental code) integration with the Rosetta design framework and thus demonstrate how one might integrate our method with a very wide variety of design tasks including protein interface antagonist design, protein interface engraftment, enzyme design, peptidomimetic design and the engraftment of complex metal binding sites onto target proteins (Leaver-Fay *et al.*, 2011; Lao *et al.*, 2014). The computational efficiency of our algorithm also enables new approaches where geometric matching is integrated more tightly with design protocols (for example, integrated into inner search loops instead of simply being performed to set up initial poses or discover starting scaffolds for a design run). The code is freely available as a set of python scripts (https://github.com/JiangTian/adaptive-geometric-search-for-protein-design).

## References

[gzy028C1] Ashworth J., Havranek J.J., Duarte C.M., Sussman D., Monnat R.J., Stoddard B.L. and Baker D. (2006) Nature, 441, 656–659. 16738662 10.1038/nature04818 PMC2999987

[gzy028C2] Berg M., de, Cheong O., Kreveld M. and van and Overmars M. (2008) Computational Geometry Springer Berlin Heidelberg. Heidelberg, Berlin.

[gzy028C3] Bhardwaj G., Mulligan V.K., Bahl C.D. et al. (2016) Nature, 538, 329–335. 27626386 10.1038/nature19791 PMC5161715

[gzy028C4] Bonneau R., Strauss C.E., Rohl C.A., Chivian D., Bradley P., Malmström L., Robertson T. and Baker D. (2002) J. Mol. Biol., 322, 65–78. 12215415 10.1016/s0022-2836(02)00698-8

[gzy028C5] Boyken S.E., Chen Z., Groves B. et al. (2016) Science (80-), 352, 680–687. 10.1126/science.aad8865 PMC5497568 27151862

[gzy028C6] Butterfoss G.L., Yoo B., Jaworski J.N., Chorny I., Dill K.A., Zuckermann R.N., Bonneau R., Kirshenbaum K. and Voelz V.A. (2012) Proc. Natl. Acad. Sci., 109, 14320–14325. 22908242 10.1073/pnas.1209945109 PMC3437879

[gzy028C7] Chapman R.N., Dimartino G. and Arora P.S. (2004) J. Am. Chem. Soc., 126, 12252–12253. 15453743 10.1021/ja0466659

[gzy028C8] Dantas G., Kuhlman B., Callender D., Wong M. and Baker D. (2003) J. Mol. Biol., 332, 449–460. 12948494 10.1016/s0022-2836(03)00888-x

[gzy028C9] Drew K., Renfrew P.D., Craven T.W. et al. (2013) PLoS One, 8, e67051. 23869206 10.1371/journal.pone.0067051 PMC3712014

[gzy028C10] Fischer D., Nussinov R. and Wolfson H.J. (1992) In CPM ’92: Proceedings of the Third Annual Symposium on Combinatorial Pattern Matching. pp. 136–150.

[gzy028C11] Fleishman S.J., Whitehead T.A., Ekiert D.C., Dreyfus C., Corn J.E., Strauch E.M., Wilson I.A. and Baker D. (2011) Science (80-), 332, 816–821. 10.1126/science.1202617 PMC3164876 21566186

[gzy028C12] Guichard G. and Huc I. (2011) Chem. Commun., 47, 5933. 10.1039/c1cc11137j 21483969

[gzy028C13] Holm L. and Laakso L.M. (2016) Nucleic Acids Res., 44, W351–W355. 27131377 10.1093/nar/gkw357 PMC4987910

[gzy028C14] Hsin K., Sheng Y., Harding M.M., Taylor P. and Walkinshaw M.D. (2008) J. Appl. Crystallogr., 41, 963–968.

[gzy028C15] Jiang L., Althoff E.A., Clemente F.R. et al. (2008) Science (80-), 319, 1387–1391. 10.1126/science.1152692 PMC3431203 18323453

[gzy028C16] Kuhlman B., Dantas G., Ireton G.C., Varani G., Stoddard B.L. and Baker D. (2003) Science (80-), 302, 1364–1368. 10.1126/science.1089427 14631033

[gzy028C17] Lao B.B., Drew K., Guarracino D.A., Brewer T.F., Heindel D.W., Bonneau R. and Arora P.S. (2014) J. Am. Chem. Soc., 136, 7877–7888. 24972345 10.1021/ja502310r PMC4353027

[gzy028C18] Leaver-Fay A., Tyka M., Lewis S.M. et al. (2011) Methods Enzymol., 487, 545–574. 21187238 10.1016/B978-0-12-381270-4.00019-6 PMC4083816

[gzy028C19] Mandell D.J., Coutsias E.A. and Kortemme T. (2009) Nat. Methods, 6, 551–552. 19644455 10.1038/nmeth0809-551 PMC2847683

[gzy028C20] Molski M.A., Goodman J.L., Chou F.C., Baker D., Das R. and Schepartz A. (2013) Chem. Sci., 4, 319–324.

[gzy028C21] North B., Lehmann A. and Dunbrack R.L. Jr. (2011) J. Mol. Biol., 406, 228–256. 21035459 10.1016/j.jmb.2010.10.030 PMC3065967

[gzy028C22] Nussinov R. and Wolfson H. (1991) Proc. Natl. Acad. Sci., 88, 10495–10499. 1961713 10.1073/pnas.88.23.10495 PMC52955

[gzy028C23] Pacella M.S., Koo D.C.E., Thottungal R.A. and Gray J.J. (2013) Methods Enzymol., 532, 343–366. 24188775 10.1016/B978-0-12-416617-2.00016-3 PMC4020438

[gzy028C24] Röthlisberger D., Khersonsky O., Wollacott A.M. et al. (2008) Nature, 453, 190–195. 18354394 10.1038/nature06879

[gzy028C25] Shin S.B.Y., Yoo B., Todaro L.J. and Kirshenbaum K. (2007) J. Am. Chem. Soc., 129, 3218–3225. 17323948 10.1021/ja066960o

[gzy028C26] Stein A. and Kortemme T. (2013) PLoS One, 8, e63090. 23704889 10.1371/journal.pone.0063090 PMC3660577

[gzy028C27] Tošovská P., Arora P.S., Tosovská P. and Arora P.S. (2010) Org. Lett., 12, 1588–1591. 20196543 10.1021/ol1003143

[gzy028C28] Wallace A.C., Borkakoti N. and Thornton J.M. (2008) Protein Sci., 6, 2308–2323. 10.1002/pro.5560061104 PMC2143595 9385633

[gzy028C29] Watkins A.M. and Arora P.S. (2015) Eur. J. Med. Chem., 94, 480–488. 25253637 10.1016/j.ejmech.2014.09.047 PMC4362920

[gzy028C30] Yoo B., Shin S.B.Y., Huang M.L. and Kirshenbaum K. (2010) Chem. A Eur. J., 16, 5527–5537. 10.1002/chem.200903549 20414912

[gzy028C31] Zuckermann R.N., Kerr J.M., Moosf W.H., Kent S.B.H.H. and Moos W.H. (1992) J. Am. Chem. Soc., 114, 10646–10647.

